# Simultaneous Expression of Cancer Stem Cell-Like Properties and Cancer-Associated Fibroblast-Like Properties in a Primary Culture of Breast Cancer Cells

**DOI:** 10.3390/cancers6031570

**Published:** 2014-07-31

**Authors:** Mami Ishikawa, Takahiro Inoue, Takuma Shirai, Kazuhiko Takamatsu, Shiori Kunihiro, Hirokazu Ishii, Takahito Nishikata

**Affiliations:** 1 Frontiers of Innovative Research in Science and Technology (FIRST), Konan University, Kobe 650-0047, Japan; E-Mails: m1461001@center.konan-u.ac.jp (M.I.); m1461002@center.konan-u.ac.jp (T.I.); sp091023@center.konan-u.ac.jp (T.S.); kazuhiko.takamatsu@jp.astellas.com (K.T.); m1361007@center.konan-u.ac.jp (S.K.); dp161001@center.konan-u.ac.jp (H.I.); 2 Frontier Institute for Biomolecular Engineering Research (FIBER), Konan University, Kobe 650-0047, Japan

**Keywords:** cancer-associated fibroblast, cancer stem cell, epithelial-mesenchymal transition, HIF1A, glycolysis

## Abstract

The importance of cancer-associated fibroblasts (CAFs) in cancer biology has been recently highlighted owing to their critical roles in cancer growth, progression, metastasis, and therapeutic resistance. We have previously established a primary culture of breast cancer cells, which showed epithelial-mesenchymal transition and cancer stem cell-like properties. In this study, we found that the primary culture also showed CAF-like properties. For example, hypoxia inducible factor 1α (HIF1A) and its downstream genes, nuclear factor-kappa B2 (NF-κB2) and BCL2/adenovirus E1B 19 kd-interacting protein 3 (BNIP3), and many enzymes involved in glycolysis, such as GAPDH, LDH, PGAM1, and PKM2, were highly overexpressed in the primary culture. Moreover, media conditioned with the primary culture cells enhanced the growth of breast cancer cells. Similar to previous CAF studies, this enhancement suggested to be occurred through fibroblast growth factor signaling. This MCKH primary culture cell, which showed simultaneous expression of tumorigenic and CAF properties, offers a unique experimental system for studying the biology of CAFs.

## 1. Introduction

Recent progress in targeted cancer therapy has revealed the importance of intratumoral heterogeneity. The presence of multiple genotypically and/or phenotypically distinct cell subpopulations within a single tumor causes therapeutic resistance and poor prognosis of cancer [1]. More recently, the complexity of cancers has been associated with the heterogeneity of the tumor microenvironment, including neoplastic cells and their surrounding stromal cells [2]. In solid tumors, stromal cells contain a variety of mesenchymal cells, such as fibroblasts, myofibroblasts, pericytes, and endothelial cells, and a variety of inflammatory cells [3].

Among the stromal cells, cancer-associated fibroblasts (CAFs) play pivotal roles in cancer growth, progression, metastasis, and therapeutic resistance [4]. CAFs produce paracrine growth factors, proteolytic enzymes, and extracellular matrix (ECM) components and interact with neoplastic cells [5]. Changes in the tumor microenvironment are due not only to different mutations within tumor cells but also to the complex interplay between diverse tumor cell types and CAFs [6]. In order to tackle the therapeutic difficulties of cancers, it is first important to understand the mechanisms involved in this changing tumor microenvironment, and hence, to reveal the characteristics of CAFs.

In our previous study, we established a primary culture of breast cancer cells, designated as MCKH (mammary carcinoma from KH), which exhibited a mesenchymal morphology and showed epithelial-mesenchymal transition and cancer stem cell characteristics [7]. In the present study, we conducted more extensive analysis of this primary culture and revealed evidence of a CAF phenotype. The implications of the simultaneous expression of tumorigenic and CAF phenotypes are discussed herein.

## 2. Results and Discussion

### 2.1. Gene Expression Profile of MCKH Cells

The gene expression profile of MCKH cells was analyzed. Total RNAs extracted from extirpated normal and malignant tissues, CRL8798, and MCKH primary cultured cells were analyzed with Agilent SurePrint G3 Human Gene Expression 8 × 60 K. Expression profiles were determined as fold changes compared to expression levels in normal tissue. The results are summarized in Table 1.

Recent studies have revealed some molecular markers of CAFs, including α-smooth muscle actin [8], collagens [9], and metallopeptidases [10]. In MCKH cells, smooth muscle actin α2 (SACTA2) and type I collagen α2 (COLA1) were up-regulated by 2.1-fold and 35.3-fold, respectively, and matrix metallopeptidase 1 (MMP1) was considerably up-regulated, by more than 2900-fold. Other previous studies also suggested the up-regulation of growth factors such as fibroblast growth factor (FGF), transforming growth factor-β (TGF-β), and vascular endothelial growth factor (VEGF) [2,11] as CAF markers. In this study, the expression of basic FGF (FGF2), TGF-β1, and VEGFA was markedly up-regulated. In addition, *v*-akt murine thymoma viral oncogene homolog 1 (AKT1), angiopoietin 1 (AGPT1), and acidic FGF (FGF1) expression was also up-regulated. These alterations in reliable CAF marker genes suggested that MCKH cells are indeed CAFs.

**Table 1:** Expression profiles of CAF-related genes in MCKH cells.

Gene name		Gene	malignant tissue *		CRL8798 *	MCKH *	
transcription factors							
HIF1A		hypoxia inducible factor 1, α subunit	1.28 ± 0.16		3.93 ± 0.16	9.40 ± 1.30	
NFKB2		NF-κB2	1.04 ± 0.12		4.25 ± 0.50	2.07 ± 0.30	
BNIP3		BCL2/adenovirus E1B interacting protein 3	0.77 ± 0.02		11.72 ± 0.23	20.85 ± 0.72	
glycolytic enzymes							
GAPDH	glyceraldehyde-3-phosphate dehydrogenase		1.08 ± 0.02		8.11 ± 0.23	16.78 ± 0.47	
LDHA	lactate dehydrogenase A		1.05 ± 0.05		21.22 ± 3.08	32.17 ± 5.40	
PGAM1	phosphoglycerate mutase 1		1.3 ± 0.50		10.84 ± 0.49	17.22 ± 2.79	
PGK1	phosphoglycerate kinase 1		1.35 ± 0.04		10.83 ± 0.19	15.67 ± 0.62	
ALDOA	aldolase A, fructose-bisphosphate		1.81		5.95	8.39	
ENO1	enolase 1, α		0.58		12.00	10.15	
PKM2	pyruvate kinase, muscle		1.31		11.59	18.56	
TPI1	triosephosphate isomerase 1		1.06		9.04	13.87	
transporters							
SLC16A1	monocarboxylic acid transporter 1		0.97		14.34	12.03	
SLC16A3	monocarboxylic acid transporter 4		5.44		116.02	136.50	
SLC1A5	neutral amino acid transporter		0.50		4.16	25.87	
others							
COLA2	collagen, type I, α2			26.20 ± 1.70	0.0006 ± 0.0002		35.32 ± 2.13
MMP1**	matrix metallopeptidase 1			1.05 ± 0.55	52.62 ± 19.16		2942.30 ± 1107.47
CAV1	caveolin 1, caveolae protein			0.28 ± 0.02	4.17 ± 0.36		0.95 ± 0.05
CAV2	caveolin 2			0.34 ± 0.03	4.64 ± 1.40		2.31 ± 0.67
AKT1	v-akt murine thymoma viral oncogene homolog 1			1.33	2.22		3.22
ANGPT1	angiopoietin 1 (ANGPT1)			0.63	0.07		6.59
FGF1	fibroblast growth factor 1 (acidic)			0.66 ± 0.41	0.53 ± 0.68		2.49 ± 0.97
FGF2	fibroblast growth factor 2 (basic)			0.22 ± 0.01	0.98 ± 0.05		9.38 ± 0.67
TGFB1**	transforming growth factor, β1			3.59 ± 0.20	6.31 ± 0.28		10.04 ± 0.54
VEGFA	vascular endothelial growth factor A			0.86 ± 0.01	1.96 ± 0.33		8.88 ± 2.73
CXCL12	chemokine (C-X-C motif) ligand 12			0.20 ± 0.005	0.0003 ± 0.0001		0.03 ± 0.002
CXCR4	chemokine (C-X-C motif) receptor 4			2.87 ± 0.07	0.003 ± 0.0003		0.008 ± 0.0008
SACTA2	actin, α2, smooth muscle			0.43	0.04		2.10

* Expression profiles were calculated as fold-changes compared to expression in normal tissue. Each value represents the average of more than two features from the gene chip. Data obtained from more than three features are presented with their SDs; ** These data have already been reported in our previous paper [7].

The oxygen-responsive hypoxia-inducible factor 1α (HIF1A) gene is overexpressed in a variety of carcinomas and their metastases [12]. HIF1A and its downstream target genes, including nuclear factor of κ light polypeptide gene enhancer in B-cells (NF-κB) and BCL2/adenovirus E1B interacting protein 3 (BNIP3), are also activated in CAFs [13]. Consequently, CAFs up-regulate the expression of glycolytic enzymes and glucose transporters [14]. HIF1A expression in MCKH cells was 9.4-fold higher than that in normal tissue. This HIF1A up-regulation was even higher than that observed in tumor tissue and a normal mammary gland breast epithelial cell line. Moreover, NF-κB and BNIP3 were also up-regulated in MCKH cells. Thus, MCKH cells show up-regulated expression of glycolytic enzymes and glucose transporters, several of which were up-regulated by more than 10-times compared to normal tissue. Some were drastically activated; for example, expression levels of lactate dehydrogenase A (LDHA) and monocarboxylic acid transporter 4 (SLC16A3) were 32-fold and 136-fold higher than those in normal tissue, respectively. These results provided compelling evidence that MCKH cells show the “reverse Warburg effect [14]”.

Another established CAF marker is down-regulation of caveolin-1 (CAV1) [14]. However, in this study, CAV1 expression was not changed. In addition, some previous reports suggested the up-regulation of stromal cell-derived factor 1 (SDF1)/chemokine (C-X-C motif) ligand 12 (CXCL12) signaling and chemokine (C-X-C motif) receptor 4 (CXCR4) as a reliable markers for CAFs [10,11]. However, in the present study, both CXCL12 and CXCR4 were strikingly down-regulated; thus, these results did not support MCKH cells as CAFs.

In order to find abnormalities in cell cycle control, we evaluated the expression of cell cycle-controlling genes (Table 2). Many cyclin and cyclin-dependent kinase genes were up-regulated, suggesting that MCKH cells were actively proliferating. On the other hand, cyclin-dependent kinase inhibitor (CKI) genes were also up-regulated. Overexpression of CKI genes, for example, CDKN1A (p21 or Cip1), results in cell cycle exit and activation of the differentiation program [15]. In the present study, many CKI genes, including CDKN1A, were also up-regulated, suggesting that MCKH cells were differentiated. Moreover, expression levels of TP53 (p53) and retinoblastoma 1 (RB1) were not significantly changed in this study. On the basis of these results, it was difficult to determine the state of cell cycle control, proliferation or differentiation, of MCKH cells.

### 2.2. CAF Properties of MCKH Cells

**Figure 1:**
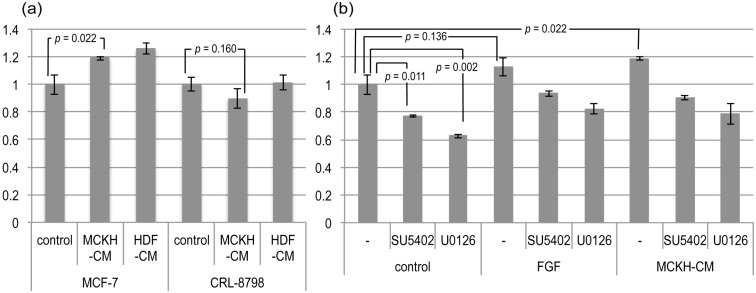
The effects of MCKH- and HDF-conditioned media (CM). (**a**) MCF-7, a human breast adenocarcinoma cell line, and CRL-8797, a normal mammary gland breast epithelial cell line, were cultured in freshly prepared MCF medium (control), MCKH-CM, and HDF-CM. Cell proliferation was assessed by using a 3-(4,5-dimethylthiazolyl-2)-2,5-diphenyltetrazolium bromide (MTT) method and evaluated relative to proliferation in the control medium. Some *p*-values of the *t*-tests are represented; (**b**) The effects of tyrosine-kinase inhibitors on the activity of MCKH-CM to MCF-7. MCF-7 was cultured in the control medium, FGF-fortified medium, and MCKH-CM, with (SU5402, U-126) or without (−) inhibitors. Cell proliferation was assessed using an MTT method and evaluated relative to proliferation in the control medium without inhibitors.

The most convincing evidence for the CAF property is enhancement of tumor growth. The human breast adenocarcinoma cell line MCF-7 and the normal mammary gland breast epithelial cell line CRL-8797 were cultured in MCKH- and human dermal fibroblast (HDF)-conditioned media (CM). As shown in Figure 1a, MCKH-CM significantly (*p* = 0.022) enhanced the proliferation of MCF-7 cells, but not of CRL-8797 cells. This result supported the CAF property of MCKH cells. However, similar enhancement of tumor growth was also observed with HDF-CM.

The enhancement of tumor growth by CAFs is suggested to be mediated by FGF signaling [2,16]. In order to determine the relationship between MCKH-CM activity and FGF signaling, MCF-7 cells were treated with FGF and/or tyrosine-kinase inhibitors (SU5402, U-0126). Treatment of SU5402 and U-0126 in the control medium decreased the proliferation of MCF-7 cells, suggesting that these inhibitors inhibited some growth signals activated by the serum contained in the control medium. The MCKH-CM was significantly enhanced the proliferations of MCF-7 even with inhibitors, SU5402 and U0126 (*p* = 0.0003 and *p* = 0.04, respectively). Although the enhancement of proliferation of MCF-7 by adding the FGF to the normal medium was not significant in this experiment (*p* = 0.136), the effects of MCKH-CM were very similar to those of FGF (Figure 1b). It seems likely that, at least, part of MCKH-CM activity is dependent on FGF signaling.

**Table 2:** Expression profiles of cell-cycle-related genes in MCKH cells.

Gene name	Gene	malignant tissue *	CRL8798 *	MCKH *
telomerase reverse transcriptase				
TERT	TERT	1.05 ± 0.22	1.13 ± 0.25	0.82 ± 0.33
cyclins				
CCNA2	cyclin A2	1.97 ± 0.83	10.27 ± 4.05	7.92 ± 3.06
CCNB1	cyclin B1	0.86 ± 0.15	6.83 ± 1.37	5.72 ± 1.43
CCND1	cyclin D1	3.23 ± 0.11	5.79 ± 0.30	7.46 ± 0.54
CCNE1	cyclin E1	1.70	10.10	4.67
cyclin-dependent kinases				
CDK1	CDK1	1.23 ± 0.30	20.13 ± 3.86	5.36 ± 1.18
CDK3	CDK3	1.50	1.74	2.88
CDK5	CDK5	2.06	5.98	6.08
CDK7	CDK7	1.07	3.34	2.72
cyclin-dependent kinase inhibitors				
CDKN1A **	p21, Cip1	3.46	7.81	19.86
CDKN2A	p16, inhibits CDK4	5.36 ± 1.79	5.10 ± 1.80	23.37 ± 10.36
CDKN2B	p15, inhibits CDK4	4.69	7.66	34.42
CDKN3	CDKN3	2.90 ± 0.75	9.10 ± 1.87	14.23 ± 7.23
others				
TP53 **	p53	0.85 ± 0.24	2.32 ± 0.71	1.54 ± 0.52
MDM2	MDM2	2.44 ± 0.67	4.21 ± 0.74	2.61 ± 0.57
RB1 **	retinoblastoma 1	1.15 ± 0.06	1.00 ± 0.04	0.84 ± 0.05

* Expression profiles were calculated as fold-changes compared to expression in normal tissue. Each value represents the average of more than two features from the gene chip. Data obtained from more than three features are presented with their SDs; ** These data have already been reported in our previous paper [7].

### 2.3. What Are MCKH Cells?

The results of this study indicated that MCKH cells show both tumor-like and stroma-like properties simultaneously. One possibility to explain this result is that MCKH primary culture represents a heterogeneous cellular population. Although, MCKH cells showed homogeneous fibroblastic morphology, and did not contain columnar-shaped epithelial-like cells, further works, such as establishment, cloning, and characterization of hTERT (human telomerase reverse transcriptase)-immortalized MCKH cell line, were needed to clarify the homogeneity. Even if the MCKH was a heterogeneous population, MCKH represents a particularly unique phenotype, which bears both tumorigenic and CAF-like properties simultaneously. This primary culture, MCKH, offers a unique experimental system for studying the biology of CAFs.

## 3. Experimental 

### 3.1. Cells and Cell Culture

The chemically transformed normal mammary gland breast epithelial cell line CRL-8798 was obtained from the American Type Culture Collection (Manassas, VA, USA), and the HDF and human breast adenocarcinoma cell line (MCF-7) were purchased from Summit Pharmaceuticals (Tokyo, Japan). The primary culture of breast cancer cells (MCKH) was established previously [7]. MCKH cells were cultured in MCF medium [Earle’s Eagle minimum essential medium; 1 mM sodium pyruvate, 10 μg/mL bovine insulin, 1× non-essential amino acids (Sigma, St. Louis, MO, USA), 10% fetal bovine serum (EuroClone, Milano, Italy)].

### 3.2. Gene Expression Profiles

Total RNAs of extirpated tissues and cultured cells were extracted with RNeasy (Qiagen, Tokyo, Japan) according to the manufacturer’s instructions. The gene expression profiles were analyzed with Hokkaido System Science (Sapporo, Japan) using the SurePrint G3 Human GE Microarray Kit 8 × 60 k (Agilent, Santa Clara, CA, USA). These data were compared to those obtained from normal tissues adjacent to the tumor, which were extirpated from the same patient. The patient provided written informed consent, and this study was approved by the Human Ethics Committee of Konan University (11-02).

### 3.3. CAF Assays

MCF-7 and CRL-8798 cells were cultured with MCKH- or HDF-CM. CM was obtained from the 24-h confluent cultures of MCKH or HDF cells with serum-starved (3%) MCF medium, and was used freshly. MCF-7 and CRL-8798 cells were cultured on 24-well plates for 48 h with CM or serum-starved (3%) MCF medium. During this culture period, culture media were replenished once. The cell proliferation rate was measured using the 3-(4,5-dimethylthiazolyl-2)-2,5-diphenyltetrazolium bromide (MTT) method. The difference between the peak absorbance at 570 nm and the minimal absorbance at 630 nm was calculated.

### 3.4. Inhibition Assays

In order to determine whether recombinant human basic FGF (Wako, Osaka, Japan; 067-04031) could mimic the effect of CM, FGF was added to the culture medium at a final concentration of 10 ng/mL. SU5402 (3-[4-methyl-2-(2-oxo-1,2-dihydro-indol-3-ylidenemethyl)-1*H*-pyrrol-3-yl]-propionic acid; Sigma; SML0443) is a fibroblast growth factor receptor (FGFR)-specific tyrosine kinase inhibitor, and is potent in inhibiting VEGFR and PDGFR [17]. U0126 (1,4-diamino-2,3-dicyano-1,4-bis[2-aminophenylthio]butadiene; Sigma; U120) is a highly selective inhibitor of MEK1 and MEK2. MEK1/2 is activated by a wide variety of growth factors, including FGF, PDGF, and EGF, and cytokines, as well as by membrane depolarization and calcium influx [18]. Both SU5402 and U1206 were added at a final concentration of 10 μM.

## 4. Conclusions

MCKH cells showed a phenotype intermediate between tumor and CAF cells. In other words, MCKH cells showed both tumor-like and stroma-like properties simultaneously. This unique property of MCKH cells might suggest an intriguing relationship between tumors and stroma. Furthermore, these cells provide clues for understanding the microenvironment of cancer, and can offer insight into the development of novel cancer therapies.
